# Comparative analysis of protein interaction networks reveals that conserved pathways are susceptible to HIV-1 interception

**DOI:** 10.1186/1471-2105-12-S1-S19

**Published:** 2011-02-15

**Authors:** Xiaoning Qian, Byung-Jun Yoon

**Affiliations:** 1 Department of Computer Science and Engineering, University of South Florida, Tampa, FL, USA; 2 Department of Electrical and Computer Engineering, Texas A&M University, College Station, TX, USA

## Abstract

**Background:**

Human immunodeficiency virus type one (HIV-1) is the major pathogen that causes the acquired immune deficiency syndrome (AIDS). With the availability of large-scale protein-protein interaction (PPI) measurements, comparative network analysis can provide a promising way to study the host-virus interactions and their functional significance in the pathogenesis of AIDS. Until now, there have been a large number of HIV studies based on various animal models. In this paper, we present a novel framework for studying the host-HIV interactions through comparative network analysis across different species.

**Results:**

Based on the proposed framework, we test our hypothesis that HIV-1 attacks essential biological pathways that are conserved across species. We selected the *Homo sapiens* and *Mus musculus* PPI networks with the largest coverage among the PPI networks that are available from public databases. By using a local network alignment algorithm based on hidden Markov models (HMMs), we first identified the pathways that are conserved in both networks. Next, we analyzed the HIV-1 susceptibility of these pathways, in comparison with random pathways in the human PPI network. Our analysis shows that the conserved pathways have a significantly higher probability of being intercepted by HIV-1. Furthermore, Gene Ontology (GO) enrichment analysis shows that most of the enriched GO terms are related to signal transduction, which has been conjectured to be one of the major mechanisms targeted by HIV-1 for the takeover of the host cell.

**Conclusions:**

This proof-of-concept study clearly shows that the comparative analysis of PPI networks across different species can provide important insights into the host-HIV interactions and the detailed mechanisms of HIV-1. We expect that comparative multiple network analysis of various species that have different levels of susceptibility to similar lentiviruses may provide a very effective framework for generating novel, and experimentally verifiable hypotheses on the mechanisms of HIV-1. We believe that the proposed framework has the potential to expedite the elucidation of the important mechanisms of HIV-1, and ultimately, the discovery of novel anti-HIV drugs.

## Background

Acquired immune deficiency syndrome (AIDS), one of the most destructive pandemics in recorded history according to the statistics from the World Health Organization (WHO) [1], has killed more than 25 million people since it was first recognized in 1981. Human immunodeficiency virus type one (HIV-1) has been found to be the causative pathogen of AIDS [2,3]. HIV-1 is a lentivirus, a slow retrovirus that is responsible for long-duration illness with a long incubation period. HIV-1 has 9 genes which encode up to 19 proteins due to post-translational cleavage [4]. By reverse transcription from viral RNA to host-integrable DNA, the virus can become active and replicate to cause rapid T cell depletion, immune system collapse, and opportunistic infections that mark the advent of AIDS [5].

Although advances in antiviral therapy and management of opportunistic infection for AIDS have remarkably improved the general health, the expensive cost and adverse effects of the available drugs have motivated many researchers to explore novel avenues to anti-HIV-1 drug discovery. With the increasing coverage of HIV-1 and human protein interactions in the literature [6-11], a human/HIV-1 interactome has been created [12], which can play a critical role in better understanding the virology and pathology of this infectious disease and developing new therapeutics. In addition to this, the availability of large-scale biological networks, including protein-protein interaction (PPI) networks, has led to the introduction of systems biology approaches for novel HIV-1 drug discovery [13,14]. In [13], Balakrishnan et al. proposed to find alternative pathways to circumvent the HIV-1 intercepted pathways based on the efficiency and robustness of biological processes. The main goal was to generate new hypotheses regarding HIV-1 targeted pathways and their effects on various molecular functions, which will help us better understand the mechanisms of HIV-1 takeover of the host cell and find ways to circumvent it. The study was based on curated signal transduction pathways obtained from multiple pathway databases. One practical problem of this pathway-based approach is that the currently known pathways cover only a limited number of human proteins, hence it may exclude important HIV-1 targets from the analysis. Moreover, many curated pathways in public databases overlap with each other, which may introduce bias in the analysis. On the other hand, Lin et al. [14] proposed comparative studies of host-virus protein interactions across human (*Homo sapiens*) and other animal models that may be invaded by similar lentiviruses that cause immunosuppression or immunoproliferation, including three mammalian species: chimpanzee (*Pan troglodyte*), rhesus macaque (*Macaca mulatta*), and mouse (*Mus musculus*). All these animal models have been extensively studied to understand the HIV-1 host-virus interplay [15,16]. Comparative studies of host-virus interactions may provide new insights into why different species have different susceptibility to HIV-1, which may lead to the development of potential therapeutics in the long run.

Motivated by these works, we propose a novel framework for studying human/HIV-1 interactions, based on comparative analysis of the human PPI network and the PPI network of other species that are susceptible to lentivirus invasion. It has been shown that the comparative analysis of PPI networks of different species can identify conserved pathways that carry essential cellular functionalities [17-36]. Furthermore, HIV-1 has to be a “minimalist” in order to survive, and for this reason, it has been believed to target these essential pathways that are conserved across species [13,37]. As a result, the comparative analysis of PPI networks may be used to generate new hypotheses that will be useful in improving our understanding of the mechanisms of HIV-1 takeover of the host cell, and ultimately, for developing effective therapeutics for AIDS.

## Results and discussion

### Conserved pathways are susceptible to HIV-1 attacks

Our main goal in this paper is to validate the following hypothesis:

“Essential biological pathways that are conserved across different species that are susceptible to lentiviruses, have a high probability of being intercepted by HIV-1.”

**Figure 1:**
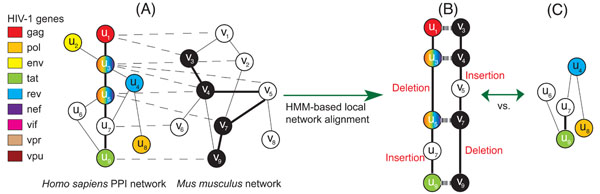
Overview of the proposed approach: (A) Illustration of the *Homo sapiens* and *Mus musculus* PPI networks along with HIV-1 interactions. The dashed line that connects two nodes *u_i_* and *v_j_* indicates that the corresponding proteins are orthologous. The solid lines represent protein-protein interactions. In the *Homo sapiens* network, the proteins are colored based on the HIV-1 proteins that can bind to them. Proteins with multiple colors are susceptible to multiple HIV-1 proteins, while proteins with no color have no known interactions with HIV-1 proteins. Note that the *Mus musculus* network is not colored. (B) The top-scoring alignment between two similar paths **u** and **v**. Colored nodes represent matched proteins. (C) An example of a randomly extracted pathway in the *Homo sapiens* network.

To validate the above hypothesis, we first aligned the *Homo sapiens* PPI network with the *Mus musculus* PPI network to find conserved pathways (Figure 1). The *Mus musculus* network was chosen as it is the largest animal network under lentivirus study that is available from public databases. This will allow us to reduce the bias that may arise from using smaller networks. For identifying conserved pathways, we used a local network alignment method based on hidden Markov models (HMMs), which we recently proposed in [36]. The HMM framework can naturally integrate both the “sequence similarity” of the proteins across different species and the “interaction reliability” between the proteins within the same PPI network into the scoring scheme for finding conserved pathways. The HMM-based local alignment method allows flexible number of consecutive insertions and/or deletions, and it can deal with a large class of path isomorphism. More importantly, the computational complexity for finding the best matching pathways grows linearly with respect to the size of each network, making it suitable for finding long conserved pathways in large PPI networks. We ran the HMM-based local alignment algorithm to find conserved pathways both with and without gaps. Since the *Mus musculus* network is still quite sparse (see **Methods**), the number of conserved pathways depends on whether we allow gaps and how long the pathways can be. Typically, we have fewer conserved pathways when we search for long pathways with no gaps allowed.

Next, we extracted 3,000 random pathways of different sizes (*L* = 16, 32, and 64) by performing a random walk on the *Homo sapiens* network (see **Methods**). Then we compared the HIV-1 susceptibility of the conserved pathways with that of the random pathways in the *Homo sapiens* PPI network, by using the predicted human/HIV-1 interactome map in [12].

#### Number of proteins in the pathways that are intercepted by HIV-1

**Figure 2:**
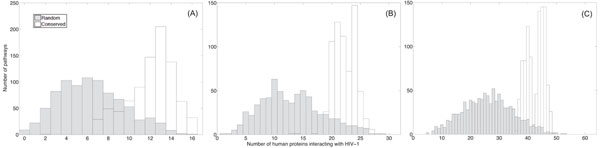
The number of proteins that interact with HIV-1 proteins in conserved pathways (with no gaps) and randomly extracted pathways. (A) The histograms for pathways of size *L* = 16. (B) The histograms for pathways of size *L* = 32. (C) The histograms for pathways of size *L* = 64.

Based on the identified conserved pathways and the randomly extracted pathways, we first computed how many proteins within each pathway can be intercepted by HIV-1, by mapping the predicted human/HIV-1 interactions in [12] onto these pathways. Figure 2 shows the histogram of the number of HIV-1 interacting proteins in conserved pathways as well as the histogram for random pathways. From the figure, there is a clear distinction between the two types of histograms. Typically, the separation between the two histograms increases with the length of the pathways. We can clearly see that highly conserved pathways are more susceptible to HIV-1 interception, in general.

**Figure 3:**
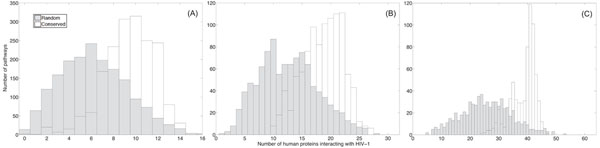
The number of proteins that interact with HIV-1 proteins in conserved pathways (with gaps) and randomly extracted pathways. (A) The histograms for pathways of size *L* = 16. (B) The histograms for pathways of size *L* = 32. (C) The histograms for pathways of size *L* = 64.

Next, we considered conserved pathways that have been identified by the HMM-based local network alignment algorithm by allowing gaps. We compared the histograms for these pathways to the histograms for random pathways. These results are shown in Fig. 3. We can see that the histograms show similar trends as in Fig. 2, but the separation between the two types of histograms is smaller in this case. This might be due to the fact that the HMM-based algorithm may find less conserved pathways when we allow gaps.

**Figure 4:**
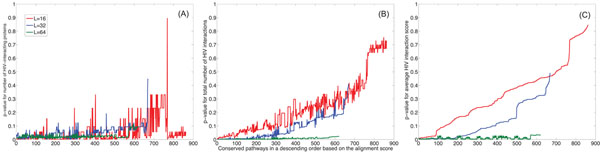
Statistical significance of the interactions between HIV-1 and the conserved pathways (with no gaps). (A) The p-values of the number of human proteins that interact with HIV-1 proteins within conserved pathways with different sizes (*L* = 16: red, *L* = 32: blue, *L* = 64: green). (B) The p-values of the total number of predicted human/HIV-1 interactions within conserved pathways. (C) The p-values for the average predicted interaction scores within conserved pathways.

To evaluate the statistical significance of the difference between conserved and random pathways, we estimated the p-value of the number of HIV-1 interacting proteins for every conserved pathway. The detailed process for computing the p-values is described in **Methods**. Basically, it computes the statistical significance of the number of HIV-1 interacting proteins in each conserved pathway with respect to the baseline distribution, which is estimated from the histogram of the number of HIV-1 interacting proteins in randomly extracted pathways. Figure 4(A) shows the plot of p-values for conserved pathways of various lengths and with no gaps allowed. Conserved pathways with gaps show a similar trend (results not shown). As we mentioned earlier, the number of conserved pathways decrease as the pathway size *L* gets larger. In the figure, the conserved pathways are sorted based on their alignment scores computed by the HMM-based local alignment method [36] (see **Methods**). The alignment score reflects the degree of conservation between the aligned pathways. We can see that highly conserved pathways are generally more susceptible to HIV-1 interception. In fact, such pathways typically contain more proteins that can be intercepted by HIV-1 proteins.

#### Total number of human/HIV-1 protein interactions within one pathway

To further validate our hypothesis, we checked the total number of human/HIV-1 interactions within each pathway. Again, we used the predicted human/HIV-1 interactome in [12] to count the total number of human/HIV-1 interactions within each pathway. Figure 4(B) shows the p-value of the total number of HIV-1 interactions within every conserved pathway (with no gaps). As before, the baseline distribution was estimated using the histogram of the total number of HIV-1 interactions in randomly extracted pathways. Note that, for long conserved pathways (*L* = 64), the p-values are always below 0.03. These results show that the difference in susceptibility to HIV-1 interception between the conserved and random pathways is statistically significant.

#### HIV-1 interaction score of conserved pathways

Finally, we evaluated the HIV-1 interaction score for conserved pathways based on the scoring scheme in [12]. For this evaluation, we mapped the prediction scores of human/HIV-1 interactions onto the conserved pathways and computed their average. In a similar way, we computed the average HIV-1 interaction score for each random pathway and estimated the distribution of these average scores. Then we computed the p-values of the average prediction scores for all the conserved pathways. These p-values are shown in Fig. 4(C) for the conserved pathways with different lengths. By comparing the results in Fig. 4(C) and those shown in Fig. 4(A,B), we can clearly see that there exists considerable correlation between these results. This is especially interesting, if we consider the fact that our approach does not use any extra data except for the PPI networks, while the prediction algorithm in [12] is obtained by integrating the information from various sources, such as gene expression, domain and motif identification, tissue distribution, functional annotation, subcellular localization and human network features, and HIV-1’s mimicry of human protein binding partners.

### GO term enrichment analysis

We also performed a Gene Ontology (GO) term enrichment analysis [38] using GO::TermFinder [39]. We took the top 20 conserved pathways of size *L* = 64 and checked their GO terms. The complete enrichment analysis results can be found at [40]. Table 1 shows some of the enriched GO terms, whose adjusted p-values are smaller than 2.0*e* – 7. Examples of highly enriched GO terms include: “signaling pathway”, “signal transduction”, “cell communication”, “phosphate metabolic process”, “response to stimulus”, “response to stress”, “protein modification process”, “regulation of immune system process”, which are pathways that are widely known to be susceptible to HIV-1 interception [2,3,12,13]. There are also more specific GO terms that are enriched, such as “hemopoietic or lymphoid organ development” and “lymphocyte proliferation”. Interestingly, the pathogenesis of HIV-associated lymphomas has been conjectured to cause the complication of HIV infection as reported in [41]. From all the proteins covered by the top 20 conserved pathways, we have listed ten human proteins with the largest number of predicted HIV-1 interactions in Table 2. We also selected eight human proteins that are not known to be intercepted by HIV-1, which are shown in bold face in Table 2. We have also listed their associated ontology keywords or GO terms based on the UniProt database [42]. We note that many of these proteins are related to the aforementioned biological processes and they might be unidentified targets of HIV-1. Further study on these proteins may lead to a better understanding of the biological mechanisms of HIV-1.

**Table 1:** Selected GO terms enriched in the top 20 conserved pathways of size *L* = 64 with adjusted p-values.

Gene Ontology terms	Adjusted p-values
signaling pathway	1.39e-49
signaling	4.39e-47
signal transduction	1.97e-42
regulation of cellular process	1.14e-41
signal transmission	4.46e-41
signaling process	4.94e-41
regulation of biological process	1.46e-39
biological regulation	3.09e-37
intracellular signaling pathway	7.57e-37
intracellular signal transduction	2.56e-35
cell proliferation	8.71e-34
phosphate metabolic process	2.86e-33
system development	3.63e-31
enzyme linked receptor protein signaling pathway	9.49e-31
developmental process	6.77e-30
anatomical structure development	1.73e-29
organ development	2.16e-29
cell surface receptor linked signaling pathway	1.23e-28
response to endogenous stimulus	7.55e-27
cellular response to stimulus	3.96e-26
response to stimulus	4.21e-25
protein modification process	2.11e-24
regulation of metabolic process	1.12e-23
response to hormone stimulus	1.38e-21
cell communication	6.28e-21
regulation of biosynthetic process	1.55e-15
Ras protein signal transduction	2.85e-14
response to stress	1.65e-13
RNA biosynthetic process	5.10e-13
cellular macromolecule biosynthetic process	5.49e-13
regulation of transferase activity	1.15e-12
immune system development	2.09e-12
regulation of immune system process	2.26e-12
**hemopoietic or lymphoid organ development**	9.12e-12
hemopoiesis	4.55e-11
neurogenesis	5.75e-08
leukocyte differentiation	6.68e-08
**lymphocyte proliferation**	1.43e-07

**Table 2:** UniProt accession numbers of selected proteins in the top 20 conserved pathways of size *L* = 64 with protein names and the associated top ontology keywords and GO terms listed by the UniProt database [42].

UniProt IDs	Protein names	Gene Ontology terms
P04637	Cellular tumor antigen p53	apoptosis; host-virus interaction; DNA damage response; protein tetramerization
P17612	cAMP-dependent protein kinase catalytic subunit *α*	hormone-mediated signaling pathway; intracellular protein kinase cascade
P28482	Mitogen-activated protein kinase 1	Ras protein signal transduction; cell cycle; transcription; interspecies interaction between organisms; chemotaxis; synaptic transmission
P27361	Mitogen-activated protein kinase 3	Ras protein signal transduction; interspecies interaction between organisms
P05412	Transcription factor AP-1	SMAD protein signal transduction; positive regulation by host of viral transcription; transforming growth factor (TGF) *β* receptor signaling pathway
P06241	Tyrosine-protein kinase Fyn	T cell receptor signaling pathway; interspecies interaction between organisms
P06493	Cell division protein kinase 1	anti-apoptosis; cell division; mitosis
Q15796	Mothers against decapentaplegic homolog 2	SMAD protein complex assembly; intracellular signaling pathway; palate development; transcription; TGF *β* receptor signaling pathway
P06400	Retinoblastoma-associated protein	Cell cycle; Host-virus interaction; androgen receptor signaling pathway; myoblast differentiation
P04049	RAF proto-oncogene serine/ threonine-protein kinase	Ras protein signal transduction; cell proliferation; protein amino acid phosphorylation
**O14512**	Suppressor of cytokine signaling 7	Ubl conjugation pathway; regulation of growth; negative regulation of signal transduction
**P10721**	Mast/stem cell growth factor receptor	male gonad development; transmembrane receptor protein tyrosine; kinase signaling pathway
**P15884**	Transcription factor 4	cerebral cortex development; regulation of smooth muscle cell proliferation; transcription
**P16410**	Cytotoxic T-lymphocyte protein 4	immune response; negative regulation of regulatory T cell differentiation
**P29597**	Non-receptor tyrosine-protein kinase TYK2	intracellular protein kinase cascade; peptidyl-tyrosine phosphorylation
**Q13480**	GRB2-associated-binding protein 1	cell proliferation; epidermal growth factor receptor signaling pathway; insulin receptor signaling pathway
**Q15503**	Son of sevenless homolog 2	apoptosis; regulation of Rho protein ; signal transduction; small GTPase mediated signal transduction
**Q96TE0**	Cdk inhibitor p27KIP1	cell cycle arrest

### Results using curated human/HIV-1 protein interactions

**Figure 5:**
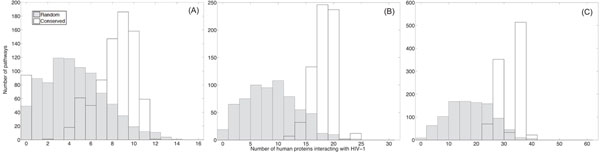
The number of proteins that interact with HIV-1 proteins based on the HPID interaction data in conserved pathways (with no gaps) and randomly extracted pathways. (A) The histograms for pathways of size *L* = 16. (B) The histograms for pathways of size *L* = 32. (C) The histograms for pathways of size *L* = 64.

**Figure 6:**
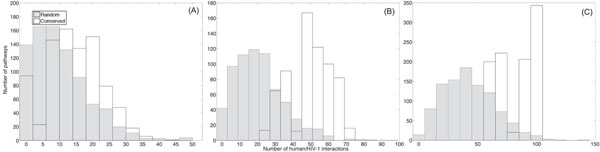
The total number of human/HIV-1 protein interactions based on the HPID interaction data in conserved pathways (with no gaps) and randomly extracted pathways. (A) The histograms for pathways of size *L* = 16. (B) The histograms for pathways of size *L* = 32. (C) The histograms for pathways of size *L* = 64.

**Figure 7:**
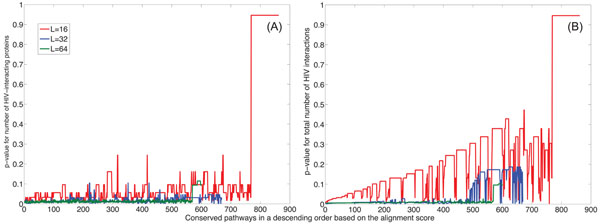
Statistical significance of the interactions between HIV-1 and human proteins within the conserved pathways (with no gaps) according to the curated interactions in HPID. (A) The p-values of the number of human proteins that interact with HIV-1 proteins within conserved pathways with different sizes (*L* = 16: red, *L* = 32: blue, *L* = 64: green). (B) The p-values of the total number of predicted human/HIV-1 interactions within conserved pathways.

In order to ensure that the obtained results are biologically meaningful, we further validate our hypothesis by testing the HIV-1 susceptibility using the curated human/HIV-1 protein interaction data in the Human Protein Interaction Database (HPID) [43]. These human/HIV-1 interactions were reported in the literature and curated by experts and can provide another supporting evidence that pathways which are conserved across species have a high probability of being attacked by HIV-1. As in our previous experiments, we counted the number of HIV-1 interacting proteins in conserved pathways (without allowing gaps) and compared it to the number of HIV-1 interacting proteins in random pathways. The resulting histograms are shown in Fig. 5. We can see that the histograms show similar trends as in Fig. 2, but the numbers of interacting human proteins are relatively smaller and the separation between the histograms of the conserved pathways and the random pathways is less significant. This is expected since the HPID curated interactions are sparser compared to the predicted interactions in [12] and smaller number of proteins in the *Homo sapiens* PPI network have been mapped with the HIV-1 interactions. We have also compared the total number of human/HIV-1 interactions in conserved pathways with that in random pathways. The resulting histograms are shown in Fig. 6. Figure 7 plots the computed p-values of both the number of the HIV-1 interacting proteins and the total number of human/HIV-1 interactions within every conserved pathway (with no gaps). Both results show that the predicted susceptibility of conserved pathways in the *Homo sapiens* PPI network and the *Mus musculus* PPI network to HIV-1 interception is statistically significant.

## Conclusions

Local network alignment can effectively identify conserved pathways that are biologically meaningful [36]. If HIV-1 is a minimalist in order to survive and therefore targets essential pathways [13], as other viruses do, it is natural to expect that essential pathways that are conserved across different species should be highly vulnerable to HIV-1 attacks. Our analysis based on comparing the *Homo sapiens* PPI network and the *Mus musculus* PPI network indicates that our conjecture is indeed true. This proof-of-concept study that we present clearly shows that the comparative network analysis of different species can provide important insights into the mechanisms of human/HIV-1 interactions. We believe that further studies based on aligning the networks of various species that are susceptible to similar lentiviruses will lead to breakthroughs in HIV research. For example, although chimpanzees are the human’s closest relative in nature, AIDS is rarely life-threatening to them [14]. Identifying the main reasons for this difference in HIV-1 susceptibility may lead to the development of novel therapeutics for this highly destructive disease. Balakrishnan et al. [13] proposed a heuristic way to search for alternative pathways that can circumvent HIV-intercepted pathways, whose ultimate goal is to identify potential drug targets. In a similar way, comparative network analysis may also be used to identify alternative pathways in the PPI network, by querying known HIV-intercepted pathways in the human PPI network. Although comparative network analysis is still at an early stage and is not yet as mature as comparative sequence analysis, it can take direct advantage of the large-scale interaction measurements that have become available these days and it has the potential to generate experimentally verifiable hypotheses on the biological mechanisms of HIV-1, which may lead to the identification of better drug targets and innovative AIDS therapeutics in the future.

## Methods

### Protein-protein interaction (PPI) networks

We have obtained both the *Homo sapiens* and *Mus musculus* protein-protein interaction (PPI) networks from the open platform NATALIE [44], managed by the Knowledge Management in Bioinformatics group of the Humboldt-Universität Berlin. Both networks were obtained from several open databases [45-50] as described in [24,51]. The *Homo sapiens* network has 34,979 interactions among 9,695 proteins, and the *Mus musculus* network has 3,116 interactions among 3, 247 proteins. The similarity between proteins in the two networks were determined based on protein sequences, protein domain information (InterProdomains) and functional annotations (GO annotations) [51-54]. Pairs of similar proteins in the two networks were identified based on a minimum protein identity threshold of *α* = 0.4 as in [24,51].

### Human-HIV interaction data

As mentioned in [13], there are several types of interactions between HIV-1 proteins and human proteins, including direct physical interactions that are reported in the literature, indirect interactions reported in the literature, and interactions that have been manually annotated by experts [12,43]. However, many HIV-1 virologists do not agree upon the majority of these interactions [13]. For this reason, we focus on the human/HIV-1 interactome in [12] in our analysis, which has been computationally predicted by integrating various types of protein features. The human/HIV-1 interactome data can be obtained from the authors’ website [55]. For further validation, we also performed similar analysis based on the curated human/HIV-1 protein interactions in HPID [43].

### Identification of conserved pathways through local network alignment

To align the *Homo sapiens* and *Mus musculus* PPI networks, we used the local network alignment algorithm based on hidden Markov models (HMMs) [35,36]. Let us represent the *Homo sapiens* and *Mus musculus* PPI networks as ***G****_h_* = (***U****,****D***) and ***G****_m_* = (***V****,****E***)*,* respectively. ***U*** = {*u_i_*} and ***V*** = {*v_i_*} represent the sets of nodes, where each node represents a protein in a given network. ***D*** = {*d_ij_*} and ***E*** = {*e_ij_*} are the sets of edges, where each edge represents the interaction between the connected proteins. For the orthologous protein pairs in the *Homo sapiens* and *Mus musculus* PPI network that have been predicted based on the method in [51], we define the node similarity score *s(u, v)* between a pair of proteins *u* ∈ ***U*** and *v* ∈ ***V*** as follows:(1)

The interaction reliability scores between two nodes are binary for both PPI networks. For example, the interaction score w_h_(u_i_,u_j_) between u_i_ and *u_j_* in the *Homo sapiens* network is defined as:(2)

The interaction reliability score *w_m_*(*v_i_, v_j_*) between *v_i_* and *v_j_* in the *Mus musculus* network is defined in a similar way.

In order to find the pathways that are conserved in both PPI networks, we first search for the best matching pair of paths **u** = *u*_1_*u*_2_ … *u_L_,* (*u_i_* ∈ ***U****)* and **v** = *v*_1_*v*_2_ … *v_L_,* (*v_i_* ∈ ***V***) in the respective networks (Fig. 1) that maximizes a predefined pathway alignment score *S*(**u***,***v**). The pathway alignment score *S*(**u***,***v**) integrates the *similarity score s*(*u_i_, v_i_*) between the aligned nodes *u_i_* and *v_i_* (1 ≤ *i* ≤ *L),* the *interaction reliability score w_h_*(*u_i_,u_i+1_*) between *u_i_* and *u_i_*_+1_ (1 ≤ *i* ≤ *L* – 1), the interaction reliability score *w_m_*(*v_j_,v_j+_*_1_) between *v_j_* and *v_j_*_+1_ (1 ≤ *j* ≤ *L* – 1), and the penalty for potential gaps in the alignment. We denote the number of nodes in a pathway as *L.* In this paper, we search for conserved pathways of size *L =* 16, 32, 64. Based on the HMM framework [35,36], we transform the problem of “finding the best matching pair of paths” to a problem of “finding the optimal pair of state sequences in the two HMMs” that jointly maximize the observation probability of a virtual path (see Fig. 1). We use two different types of settings for finding conserved pathways, where we do not allow gaps in the pathway alignment in one setting while we allow gaps in the other setting. In general, the two setting will yield different predictions. We can find the best matching pair of paths in the given networks using dynamic programming. For this purpose, we first define the score for the most probable pair of a subsequence paths of length *t* (≤ *L*) as follows:(3)

Next, we find the optimal pair of paths (u*, v*)(4)

by computing the score (3) iteratively. As discussed in [35,36], we can add auxiliary states to the HMMs that represent the PPI networks to find gapped path alignments. Instead of finding only the best matching pair of paths, we can also search for the top *k* path pairs by replacing the max operator in (4) and (3) by an operator that finds the *k* largest scores. The computational complexity of the described dynamic programming algorithm is only *O*(*kLM*_1_*M*_2_) for finding the top *k* pairs of matching paths, where *M*_1_ is the number of edges (i.e., interactions) in ***G****_h_*, and *M*_2_ is the number of edges in ***G****_m_*. Note that the computational complexity is linear with respect to each parameter *k, L, M*_1_, and *M*_2_. To avoid multiple occurrences of the same protein in the conserved pathways that are predicted by the algorithm, we incorporate a “look-back” step into each iteration of the dynamic programming algorithm [36].

### Extraction of random pathways

In order to extract random pathways from the *Homo sapiens* PPI network, we performed random walks on the network starting from a randomly selected node in network *G_h_*. We randomly walk on the network to choose a random pathway, until the size of the pathway reaches a pre-specified size *L*. During this random walk, we avoid visiting a node that has been previously visited, so that the extracted random pathway contains only distinct nodes.

### Comparison between conserved pathways and random pathways

To compare the HIV-1 susceptibility of conserved pathways with that of random pathways, we computed the following values: (1) The *number of proteins* within these pathways that have been predicted to be intercepted by at least one of the HIV-1 proteins according to the human/HIV-1 interactome in [12]. (2) The *total number of predicted human/HIV-1 protein interactions* within these pathways; (3) *The average HIV interaction scores* within pathways. We also computed the p-values of the estimated results for conserved pathways, according to the process described in the next subsection. Finally, for GO term enrichment analysis, we used an open source software called the GO::TermFinder [39].

### Computing p-values

In order to evaluate the statistical significance of the estimated results in conserved pathways, we first extract a large number of random pathways (3, 000) from the *Homo sapiens* PPI network, based on random walk. For each random pathway, we also estimate the indices of HIV-1 susceptibility (i.e., the number of human proteins intercepted by HIV-1; the total number of human/HIV-1 interactions, the average interaction scores among the proteins in each pathway). Baseline distributions of different indices are estimated from these results. We can either model the baseline distributions using Gumbel distributions [56] or simply use histograms. The latter approach was adopted in this paper. Once we have estimated the baseline distributions, we can compute the p-values of the estimated results in conserved pathways according to the estimated distributions.

## Authors' contributions

Conceived and designed the experiments: XQ, BJY. Performed the experiments: XQ. Analyzed the results: XQ, BJY. Wrote the paper: XQ, BJY.

## Competing interests

The authors declare that they have no competing interests.
